# Investigating the Socio-Economic and Livelihoods Impacts of African Swine Fever in Timor-Leste: An Application of Spatial Group Model Building

**DOI:** 10.3389/fvets.2021.687708

**Published:** 2021-11-18

**Authors:** Jared Berends, Joanita Bendita da Costa Jong, Tarni Louisa Cooper, Kanar Dizyee, Olavio Morais, Abrão Pereira, Dominic Smith, Karl M. Rich

**Affiliations:** ^1^The New Zealand Institute for Plant & Food Research Limited, Auckland, New Zealand; ^2^International Livestock Research Institute, West Africa Regional Office, Dakar, Senegal; ^3^Ministry of Agriculture and Fisheries, Government of Timor-Leste, Dili, Timor-Leste; ^4^School of Veterinary Science, The University of Queensland, Gatton, QLD, Australia; ^5^School of Agricultural and Food Sciences, The University of Queensland, St. Lucia, QLD, Australia; ^6^Menzies School of Health Research, Dili, Timor-Leste; ^7^Griffith Asia Institute, Griffith University, Brisbane, QLD, Australia; ^8^Ferguson College of Agriculture and Department of Agricultural Economics, Oklahoma State University, Stillwater, OK, United States

**Keywords:** African Swine Fever, spatial group model building, Timor-Leste, value chain, livelihoods

## Abstract

Small-scale pig farming is highly important to the economic and social status of households in Timor-Leste. The presence of an African Swine Fever (ASF) outbreak in Timor-Leste was confirmed in 2019, a major concern given that around 70% of agricultural households practice pig farming. This research used a virtual spatial group model building process to construct a concept model to better understand the main feedback loops that determine the socio-economic and livelihood impacts of the ASF outbreak. After discussing the interaction of reinforcing and balancing feedback loops in the concept model, potential leverage points for intervention are suggested that could reduce the impacts of ASF within socio-economic spheres. These include building trust between small-scale farmers and veterinary technicians, strengthening government veterinary services, and the provision of credit conditional on biosecurity investments to help restock the industry. This conceptual model serves as a starting point for further research and the future development of a quantitative system dynamics (SD) model which would allow *ex-ante* scenario-testing of various policy and technical mitigation strategies of ASF outbreaks in Timor-Leste and beyond. Lessons learned from the blended offline/online approach to training and workshop facilitation are also explored in the paper.

## Introduction

Small-scale pig farming plays a vital role within Timorese economic and social spheres. Across both urban and rural settings, over 70% of agricultural households raise pigs, with the average household keeping fewer than three pigs (1). Pigs are kept by around 114,598 households with a national herd total of 453,444 (1). The most common pig production system is an extensive scavenging system, with only a small portion of pigs raised in confined smallholder semi-intensive and intensive systems (2). Pigs are highly valued for cultural ceremonies, with pork consumption outside of these times being relatively low (3). Such is the value placed on pigs that households will continue to purchase them for cultural purposes even when they are unable to supply them from their own household farms. The significant cultural value is reflected in the high monetary price of pigs in Timor-Leste. The average herd of a small-scale farmer is valued at US$ 1200, making pigs the largest contributor to household incomes from the livestock sector (2, 3). This is a significant savings stock in a country where 70% of the population lives on less than US$ 3.20 per day (3).

Since independence in 2002, Timor-Leste has made strides toward socio-economic progress as evidenced by steady rises in nominal income per capita (US$ 508 in 2002–US$ 1237 in 2018) and the Human Development Index (0.505 in 2000–0.626 in 2018). The economy remains largely dependent on oil and gas, which accounts for around 33% of total GDP, and finances 90% of the state budget (4). Most of Timor-Leste's population of 1.2 million people are not involved in formal regular employment; instead, households depend upon multiple small livelihood activities and subsistence agriculture (4). Around 41.8% of the population live below the national poverty line with undernourishment of under-five children a persistent issue (5). As found in other Southeast Asian countries, household pig farming in Timor-Leste functions as an important livestock bank for the poor; pigs are sold during times of financial stress or to fund lumpy expenses, such as education costs.

The presence of an ASF outbreak in Timor-Leste was confirmed in September 2019. Before testing was scaled back due to COVID-19 restrictions, it had spread to eight out of 13 municipalities. Within 6 months of detection, nationwide mortalities had exceeded 50,000 pigs, around 11% of the national herd (2). Underpinning the potential for widespread socio-economic impacts of an ASF outbreak is the chronic under-investment in the veterinary sector and the important role pig farming plays in livelihoods and cultural ceremonies, particularly for the most vulnerable households sitting below or around the poverty line (3, 6).

The need for an analytical tool to evaluate the potential impact of ASF on small-scale pig producers and their livelihoods and the future opportunities to restock pig herds after an ASF outbreak motivates the use of a systems approach. We deployed a system dynamics (SD) approach to capture and model the multiple feedback effects within the pig value chain (VC) system, particularly the interactions between small-scale producers, household savings, disease outbreak, and the veterinary system. A unique advantage of SD approaches is that models of the system can be co-created with community members and other stakeholders through a well-documented process known as group model building (GMB) (7). In a recent evolution of GMB, spatial aspects and drivers of livestock systems have been incorporated within a process termed spatial group model building (SGMB), enriching the scope of information gathered through stakeholder facilitation and improving model design and outputs (8, 9).

This paper covers the process and tools used to pilot SGMB in Timor-Leste to understand the feedback loops and relationships that contribute to the socio-economic and livelihoods impacts of the recent ASF outbreak. A simple conceptual model of the socio-economic impacts of ASF within small-scale pig farming systems is presented. This concept model indicates several prospective feedback loops which drive behavior in the pig VC in Timor-Leste. Following a discussion on the interaction of reinforcing and balancing feedback loops, potential leverage points for intervention are suggested that could reduce the impacts of ASF within socio-economic spheres. Two critical innovations, one methodological and one practical, which enhance our knowledge of the livelihoods impacts of animal disease are also highlighted in the paper. First, to the research team's knowledge, participatory SD methods have not previously been used in Timor-Leste. The paper demonstrates that SGMB tools provide a simple and effective platform for VC actors to exchange perspectives and come to a common understanding on the key dynamic relationships which determine impacts in livestock systems. Second, the work in Timor-Leste piloted a hybrid online/offline form for participatory engagement given COVID-19 travel restrictions, which is elaborated upon in this paper as an example for future applications.

## Materials and Methods

### Overview of SD and SGMB Methodology

SD approaches are increasingly used to construct qualitative and quantitative models of agricultural systems and VCs (10–13). SD is a modeling and analytical paradigm developed during the mid-1950s by Professor Jay W. Forrester. At its core, SD is an approach to solving problems based on dynamic behavior in complex systems and it has since been applied in diverse fields, such as economics, public policy, environmental studies, defense, commodity cycles, and management (14). SD practitioners develop models as a means of understanding the consequences of behavior resulting from interactions and feedback between different actors and/or decisions. Within SD modeling, systems are represented by stocks, flows, converters, and feedback loops. Stocks reflect the state of the system at a given point in time, and represent, for example, an accumulation of services, goods, funds, or knowledge. Flows denote changes over time and regulate the inflow and output of goods or services from a stock, with converters determining the rate of flows over time or affecting other converters. Feedback loops are circular causalities that regulate flows through delayed circular causal (and often nonlinear) relationships among model components (15). Recently, SD models have been deployed to conduct *ex-ante* impact assessments of livestock sectors in countries such as Botswana (16, 17), Namibia (18), Indonesia (19), and Myanmar (20). This has also included previous application in Uganda in the context of measures to mitigate an ASF outbreak across VC actors (21).

The process of GMB co-creates SD models through facilitation with stakeholders in focus group discussions (7). These models provide a platform for stakeholders to jointly analyze the impacts and trade-offs of potential policy or technical interventions prior to investments being made, thus leading to a more robust decision-making process that is co-owned by the group. The SGMB process builds upon the widely used tools and techniques developed within GMB methods. GMB and SGMB sessions typically comprise of 10–15 people; larger groups slightly complicate the use of participatory GIS (Geographical Information Systems) techniques (9). These sessions act as focus group discussions and should comprise a diverse set of VC stakeholders, with balance in terms of roles and gender carefully maintained. They are facilitated by a team which typically includes a lead facilitator, assistant facilitator, note takers, a process coach who manages and supervises the team, and a lead modeler who converts focus group discussions into working SD models (7, 9). While some of these roles can be combined, a minimum of three people is needed to facilitate these sessions, with the role of the lead facilitator, note taker, and modeler always distinct. Agendas for each session are carefully planned and aim to provide a roadmap for each GMB session, guiding the facilitation team in the process, team roles and behaviors, time available, and desired outputs (22, 23). Training of the facilitation team, including mock sessions, is an integral precursor to the process with a particular focus on the team's attitudes, skills, and teamwork (7). Within SGMB, a reference group of technical experts complements the focus group discussions with VC stakeholders. The reference group provides feedback and an external reality check on the process and information collected through regular discussions, which can be through a combination of formal meetings and/or *ad hoc* interactions (emails, phone calls, etc.) (24).

Both SGMB and conventional GMB techniques lead focus group discussions through “scripts,” which are a set of guided activities aimed at achieving a specific objective in the facilitation and modeling process (23). The initial scripts in a set of GMB sessions seek to organize the process (logistics, participant invitations, etc.), introduce the approach to stakeholders, gauge participant expectations through a “Hopes-and-Fears” exercise, and introduce basic concepts of systems thinking (stocks, flows, converters, feedback loops) by using simple, practical examples. Conventional GMB sessions then move toward the facilitation of key system variables and reference modes with stakeholders (i.e., dynamic trends of behavior) (25). By contrast, SGMB sessions follow the introductory scripts with an extended participatory exercise using principles of GIS. A participatory facilitation tool, known as Layerstack, was previously developed to help facilitators and participants come to a common, visualized understanding of the system (8). Layerstack is a type of offline GIS in which plastic acetates serve as data “layers” overlaid on a base map of the region in question. Layer definitions are pre-defined by the facilitation team and can include patterns of trade, land use, socio-economic characteristics, and animal disease outbreaks. Various consumables (stickers, markers, post-it notes) are used to label spatial characteristics by participants, and reference modes and running legends are directly drawn on the edges of the map to illustrate trends in spatial variables.

From the Layerstack exercise, which typically takes place over a 90- to 120-min period, a subsequent set of scripts are implemented that identify and prioritize problems; elucidate the causes and consequences of prioritized problems; and reveal core system modules for further stakeholder-led modeling and identification of parameters and model structure (9). In previous applications, four to five SGMB sessions were held over a 6- to 8-week period culminating in the initial concept model with quantified parameters. Subsequent work by the facilitation team further refines and parameterizes the model developed with stakeholders (and informed by the reference group) over the following few months, after which a finalized quantitative SD model is presented to participants for wider feedback and refinement. Available primary and secondary data complement the process; in some cases, a rapid VC analysis using conventional techniques precedes the SGMB sessions (20, 26). The quantitative SD model is validated by stakeholders to ensure it is an accurate representation of the system, and through a series of standard tests, including ensuring parameters hold real-world meaning and the model is able to replicate historical trends [see Forrester and Senge (27)]. Following validation the model is used to conduct an *ex-ante* impact evaluation of potential intervention scenarios. The results of scenario-testing are then shared with stakeholders to support decision-making and encourage the ownership of recommendations (7).

### Research Team

Researchers from the University of Queensland (UQ) and the International Livestock Research Institute (ILRI) partnered with six staff from Veterinary Services within Timor-Leste's Ministry of Agricultural and Fisheries (MAF) and Menzies School of Health Research (MSHR) to conduct the field research. Due to COVID-19 travel restrictions, UQ and ILRI conducted online training and provided support for MAF and MSHR staff who facilitated the three face-to-face SGMB workshops in Dili, Timor-Leste with 13 participants from the pig VC. Ethical clearance (approval number 2020001543) was obtained from UQ prior to conducting the research.

### Training of the SGMB Team

Training on SD and SGMB was conducted in June 2020 and led by ILRI team members. MAF and MSHR staff participated in six initial online training sessions of 90–120 min, covering: (i) an introduction to systems thinking and SGMB; (ii) how to plan an SGMB process; and (iii) how to use key SGMB tools (Layerstack, cause and consequence mapping, and the development of concept modules). Training sessions were conducted online via Zoom (https://zoom.us/) and utilized a range of online engagement tools, such as Padlet (https://padlet.com/), Jamboard (https://jamboard.google.com/), and Vecta (https://vecta.io/). Padlet is a document storage system which allows easy access to training materials and contained links to the Jamboard and Vecta web pages. Jamboard is a web-based platform operated by Google for real-time collaboration and brainstorming, providing a simple way of replicating a whiteboard online. It allows participants to write sticky notes and link/cluster them together by color or with freehand text in a shareable fashion with others in the workshop. Vecta is a free online editor for collaborative graphics editing. It mimics the participatory GIS features of Layerstack by including a feature whereby layers of information can be overlayed on top of one another. While training activities covered critical points of SD and SGMB theory, sessions were weighted toward the use of the tactile participatory modeling tools to build the skills and confidence of MAF and MSHR staff to facilitate critical elements of upcoming SGMB sessions.

Following the formal training workshops, another two sessions were held to develop the agendas (included in this article's Supplementary Materials) for the three SGMB workshops and to conduct a practice run of participatory tools. These practice runs helped MAF, MSHR, and ILRI researchers to trial different workshop techniques, ultimately settling on a blended online and offline approach. This approach consisted of MAF and MSHR staff facilitating in-person SGMB workshops using tactile participatory tools and a virtual coaching presence from ILRI and UQ using Zoom and WhatsApp (https://www.whatsapp.com/) voice and video technologies. Additional MAF staff joined these practice sessions to act as mock workshop participants. Further one-to-one coaching sessions were held with facilitators in the days leading up to the SGMB workshops to respond to questions around facilitation techniques of participatory tools.

### SGMB Process

Given that the focus of the study was to pilot SGMB tools to develop a simple concept model, it was decided to shorten the process to three workshops. These were held at the MAF office in Dili, Timor-Leste over a 10-day period in August 2020. Workshops were scheduled to last for half a day, starting in the morning and concluding with a lunch for attendees. The MAF and MSHR team selected Tasi Tolu, a peri-urban area in Dili as the model's boundary because of the mixture of urban and rural villages and the accessibility of workshop participants. MAF and the MSHR were confident that pig farming in Tasi Tolu was broadly representative of practices throughout Timor-Leste with workshop participants recruited by MAF staff through their networks of local veterinary offices in Tasi Tolu, i.e., purposive sampling. A total of 13 participants from across the pig VC attended workshop one, which dropped to 12 for workshop two and nine for workshop three. Of the 13 participants, two were female, and while most participants identified themselves as pig farmers (9), pig traders (2) and veterinary technicians (3) also attended. The attending pig farmers were backyard producers, typically keeping between two to five hogs at any given time. Workshop dropouts came from pig trader and producer segments of the VC. SGMB workshop one and two were held on consecutive days and SGMB workshop three 9 days later which may explain the drop in attendance. Participant travel costs were reimbursed and they were provided with participation certificates from MAF.

MAF and MSHR staff facilitated the workshops, playing the key SGMB roles of lead facilitator, assistant facilitator, and note taker. Additional roles were added to the in-country team given the blended workshop approach. A liaison/translator role was established to maintain a virtual connection with the team from ILRI who fulfilled the process coach roles. The liaison/translator would translate critical elements and act as the process coaches' “voice” into the workshop. This allowed researchers from ILRI to ask further questions and provide nuanced course correction during participatory exercises. During breaks in the workshop, the process coaches were able to speak directly to the lead facilitator, providing additional feedback and encouragement. Two video links between the process coaches and the workshop were maintained by way of a broad camera link that captured the entire workshop space (via Zoom) and a second handheld camera link (via WhatsApp) through which the liaison/translator could show details of workshop outputs, such as Layerstack maps. The modeler function was undertaken by a member of the ILRI team who also acted as one of the process coaches.

The objective of the first workshop was to introduce SD and SGMB principles to workshop participants and to use Layerstack to understand the spatial dynamics of the pig VC and the socio-economic impacts of ASF. The hopes-and-fears exercise (9) at the start of the workshop helped address any concerns or misunderstandings held by participants. This proved useful in unearthing an assumption held by some attendees that the workshop was a training on ASF. These participants readily accepted the facilitator's explanation that the purpose of the workshop was to co-create a model to learn more about the socio-economic impacts of ASF. The physical Layerstack toolkit previously used to conduct participatory GIS exercises (9) was not available due to COVID-19 related postal delays. As such, the underlying A3 map of Tasi Tolu was taped to the workshop wall and plastic sheets overlaid onto it to collect the layered spatial and temporal information. Following an introduction to Layerstack, 15 min was allocated for each of the five layers that covered (i) pig production zones; (ii) key inputs and services for pig production; (iii) the movement of pigs from pig production zones to other VC nodes (i.e., villages, traders, butchers, wholesalers, retailers); (iv) other livelihood practices and their contributions to household incomes and socio-economic status; and (v) impact of ASF on livelihoods. A prioritization exercise on problems related to ASF elucidated during Layerstack was then conducted. Participants individually wrote down one key problem and after a brief summary of the problems by the facilitator, participants voted for their top problem, ultimately prioritizing (i) the lack of technical veterinary services available and (ii) the loss of household income from pig farming.

The second SGMB workshop began with a recap of these two problems and an introduction to the basic terminology of SD (stocks, flows, and converters) using the water-in-a-glass script (9). Following this, cause-and-consequence maps of the priority problems were constructed. To initiate this interaction, a plenary discussion on the nature of the problems was held, culminating in the development of reference modes on the whiteboard which included temporal and spatial characteristics of the problems. The reference mode is a visualization of the current trend and trajectory of a problem over time. It is used to help characterize and describe the problem and ensure there is consensus among participants as to its nature and evolution. Reference modes utilize “behavior over time” graphs; in this research, this consisted of drawing out the pig population over the last 10 years and the last year. Next, participants identified and discussed the problem's root causes and expanded consequences which were placed on the whiteboard. The causal relationships and key feedback loops which drive system behavior were then identified by participants by asking them to identify consequences of problems that circled back to alter original problem causes. Based on the issues and relationships identified in the cause-and-consequence maps, participants and the facilitation team selected four thematic areas that govern behavior in the pig VC system during an ASF outbreak. These thematic areas became the modules for further development using SD terminology: pig production, veterinary services, socio-cultural practices, and farm finances; ultimately acting as the concept model's boundary. The 8-day space between workshop two and three enabled the modeler from ILRI to develop simple preliminary stock and flow diagrams of these modules for expansion in the third workshop.

The aim of the third SGMB workshop was to develop simple qualitative concept modules using basic SD terminology to capture participant understanding of the relationships in the pig farming system and the impacts of ASF. Concept modules are a qualitative tool that visually represents the most critical parts of any system (i.e., a closed boundary) and capture dynamic complexity by documenting the polarity of relationships between stocks, flows, and converters, and the identification of feedback loops and time delays (14). Following a refresher on these key SD concepts, the facilitator presented the preliminary stock and flow diagrams to participants and added structure based on their feedback and responses to question prompts. These diagrams were sketched on whiteboards to allow for their iterative development and included the polarity of relationships (i.e., the direction of cause and effect relationships) and feedback loops identified. Once the structures of the individual modules were developed, they were then combined to pinpoint inter-module connections. Following the third workshop the concept model was revised by the modeler and shared with the research team for finalization.

## Results

### Overview of the Pork Value Chain

Along with the development of the concept model, the SGMB workshops helped frame the underlying problems and behaviors in the pig system in Tasi Tolu. Participants noted that there had been a steady decline in pig stocks in the target area over the last 5 years related to the application of a law that banned the free roaming of pigs in urban areas. Without the ability to let their pigs roam, pig farmers faced increased housing and feed costs. Pig feed mainly came from leftover household food and restaurant scraps. Piglets were usually purchased from within or nearby villages but there was no formal credit mechanism to help farmers restock after frequent disease outbreaks. Very few of the farmers had a relationship with the local veterinary technician (VT) and relied on traditional methods or medicine purchased from the local agricultural input supplier to maintain healthy pigs. None of the farmers present vaccinated their pigs. Farmers retained their pigs for traditional cultural purposes but also sold to neighbors and the local pork wholesale market when the household required cash. These pig sales typically comprise 20–30% of the household's yearly cash income. This supplements the other main livelihoods in the area of fishing, small livestock raising (goats, chickens, and ducks), operating small consumer supply shops, and selling of smoked fish and palm syrup. While income from other livelihoods would generally enable pig farmers to restock following a disease outbreak, the scarcity and high price of piglets and sows following the recent ASF outbreak had prevented many farmers from reinvesting. The ASF outbreak had also caused a high-level of mistrust in the system, as farmers were worried that they could not prevent or contain a future ASF outbreak nor could they verify the health of pigs and piglets flowing into their village.

The SGMB participants prioritized two main problems in the pig system that exacerbated the current situation. First, there was a lack of technical veterinary services available to pig farmers. While there is a general standard of one VT per administrative post, it was acknowledged that this is insufficient to meet the requirements of farmers, with SGMB members suggesting village-level workers were necessary. Along with a lack of human resources, existing VT lacked transportation and communication equipment to conduct regular visits to villages. Some participants noted that government revenue from pigs was low and therefore this decreased the incentive to invest in support services. The second problem identified centered on the loss of household income from pig farming. With limited access to formal financial services and high prices, farmers were unable to invest in pig farming, robbing them of a vital safety net. Hogs were often kept and sold to cover lumpy household cash requirements, such as school fees and uniforms or investments in other livelihoods, such as purchasing new fishing equipment or stock for shops. Furthermore, hogs were required for cultural ceremonies like weddings and funerals. The lack of hogs and high purchase prices further exacerbated the loss of household savings and potentially alienated households from relatives who often form a reciprocal social safety net.

### Concept Model of the Timor-Leste Pig System

**Figure 1:**
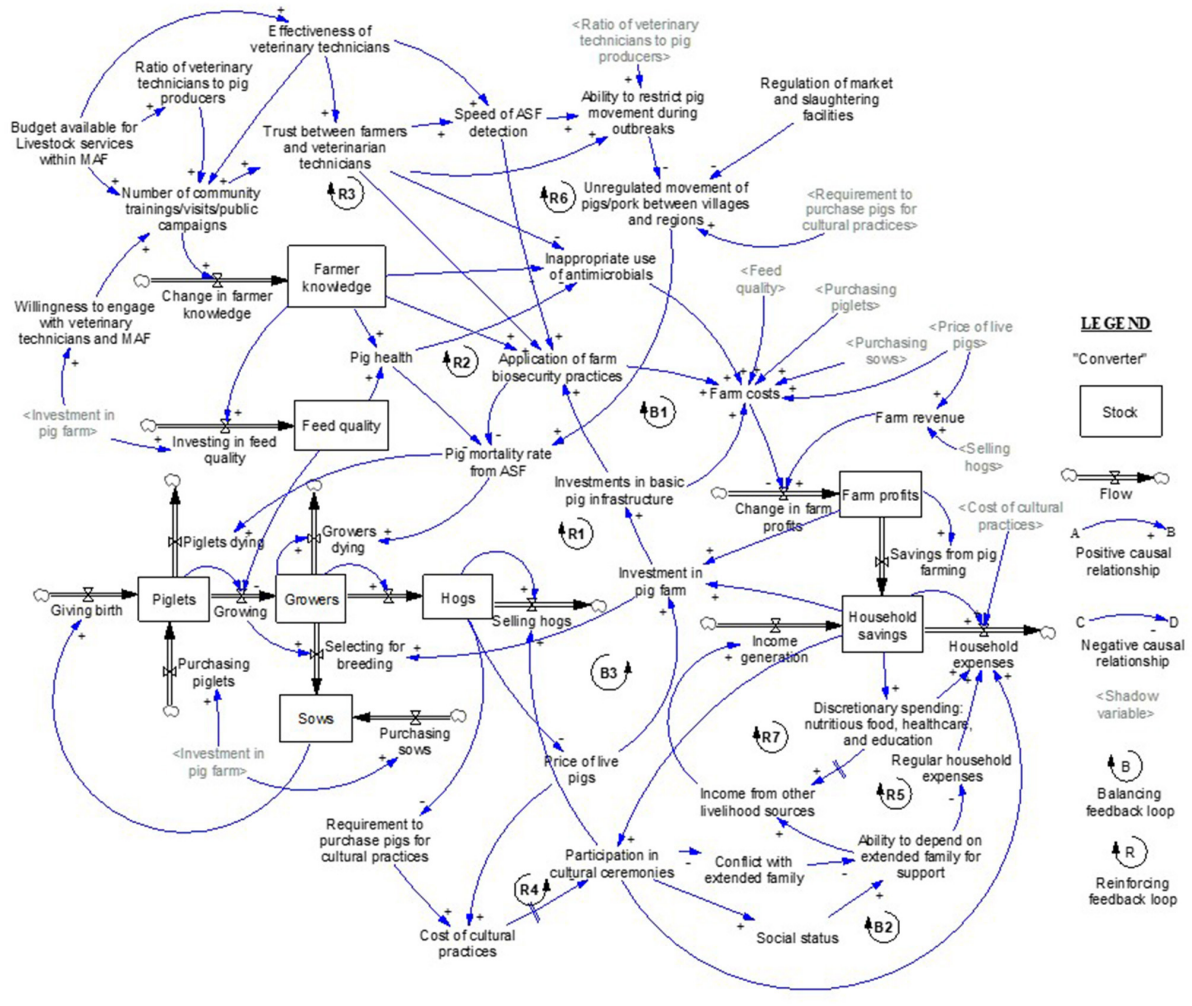
Concept model of the Timor-Leste pig system, including interactions between marketing dynamics, production practices, livelihoods and socio-cultural dimensions, farmer knowledge, and animal health infrastructure. Gray parameters are repeated “shadow” variables from the concept model. “R” indicates reinforcing feedback loops and “B” indicates balancing feedback loops in the system. Unboxed text represents key converters (also known as parameters) in the model while boxed text represents key stocks in the system and black arrows show flows into and out of these stocks. Blue arrows show critical casual relationships between stocks, flows, and converters with the + sign indicates movement in the same direction as the origin of the change and the—sign indicates movement in the opposite direction to the origin of change.

The key output of the research process is a basic concept model of the pig system in Timor-Leste, as shown in Figure 1. The concept model was developed by participants over the course of the SGMB workshops and later refined by the research team. All participants actively engaged in the model building process, though the three male veterinary technicians were the most active. Originally the concept model was to be shared with participants and other stakeholders for comments, though time limitations prevented this verification step. This concept model includes interactions between production practices, livelihood and socio-economic and cultural dimensions, farmer knowledge, and animal health infrastructure that determine system responses to an ASF outbreak.

The SGMB process identified prospective feedback loops that drive system behavior. These loops are denoted as “R,” Reinforcing or “B,” Balancing feedback loops in Figure 1. Reinforcing feedback loops amplify behavior and when activated result in either exponential growth or decay (28). In contrast, the balancing feedback loop is a self-adjusting loop that seeks to counteract and oppose change, thus balancing the system to some level of stasis or equilibrium (14). A brief explanation of the core feedback loops follows. To aid understanding of the concept model, relationships within certain loops are described in unidirectional terms, i.e., increasing or decreasing; however, all feedback loops can operate in either direction (28). A figure of each individual loop is found in the Supplementary Materials B.

#### R1: Farm Production Investments

Changes in profits alter farmer willingness to invest in pig farming, which causes farmers to expand/contract the size of their pig farms through changing the number of breeding sows and the volume of piglets purchased. This affects the number of hogs sold and leads to further increases/decreases in farm profits.

#### R2: Farm Biosecurity/Health Investments

Changes in farm profits affect investments in pig feed, infrastructure (pig pens, watering systems, etc.), and the willingness of farmers to engage with (and pay for the services of) VT and MAF staff. This in turn impacts a farmer's application of biosecurity practices and the level of pig health, altering the pig mortality rate from diseases, such as ASF. Changes in mortality rates alter the proportion of pigs dying, affecting the number of hogs sold, leading to further changes in farm profits.

#### B1: Farm Costs

Increasing investments in pig production and biosecurity/health investments lead to higher farm costs which lower profits and reductions in these investments.

#### R3: Trust

Trust between VT and farmers increases when they engage more frequently through trainings, field visits, and public awareness campaigns, and advice provided by VT increases farmer knowledge and improves pig health. As trust grows, farmers are more likely to report unexplained pig deaths to VTs, allowing earlier detection of ASF and the prompter application of farm biosecurity practices and adherence to movement restrictions between villages and regions. These lessen the pig mortality rate from ASF which results in higher farm profits and household savings, leading to higher post-ASF outbreak investments in pig production and an increased willingness of farmers to engage with VT and MAF staff. The increased trust also prevents the inappropriate use of antimicrobials, lessening farm expenditure and further increasing farm investments and trust with VTs and MAF.

#### R4: Providing Hogs for Cultural Practices

When hog numbers in a village decrease, farmers must increasingly purchase pigs for cultural purposes rather than using pigs from their own stocks. As pig stocks reduce this further inflates the price of purchasing pigs and the financial cost of cultural practices. Given the high cultural value placed on pigs, there is a delay between the rising costs of cultural practices and reduced participation in cultural ceremonies. Until this point is reached, purchasing pigs for cultural practices increases household expenditure and draws down household savings, reducing the ability of farmers to reinvest in pig farming and furthering lowering the overall number of hogs in the system.

#### B2: Reducing Participation in Cultural Practices

When household savings fall and the price of live pigs increase, at some point, households lessen their participation in cultural ceremonies involving the use of pigs or other livestock. The reduction in demand to purchase pigs for cultural ceremonies causes stocks of hogs to rise. This lowers the price of live pigs, reducing the financial costs associated with cultural practices and increasing household savings which leads households to start participating in cultural ceremonies again.

#### R5: Social Capital

When farmer participation in cultural ceremonies falls, there is a loss of face and less contact time between family members. As a result, the likelihood of misunderstanding and conflict with extended family members rises, and household social status falls, both of which decrease the ability to depend on extended family members for support. This lessens the ability of households to generate income from other livelihoods or meet regular household needs through gifts-in-kind or cash provided by extended family members. This reduces the stock of household savings and further limits the household's ability to participate in cultural ceremonies.

#### R6: Movement of Pigs

When the number of hogs in one geographic location decreases, people purchase hogs from another village/region for cultural practices, increasing the movement of pigs across the country. This growth in movement raises the rate of spread of ASF across Timor-Leste, leading to further pig deaths and a shortage of hogs.

#### R7: Poverty Spiral

As household savings decrease, the household's ability to purchase nutritious food, healthcare, and education fall which, after some time, will negatively impact their ability to generate earning, thereby further reducing household savings.

#### B3: Restocking

As the number of hogs in the system decreases the price of live pigs rise, incentivizing investment in pig farming. This increases the number of hogs in the system and diminishes the price of live pigs.

## Discussion

### Leverage Points

The concept model of the pig VC allowed the identification of potential leverage points to help mitigate the socio-economic impacts of an ASF outbreak in Timor-Leste. Leverage points are parts of the system that, when changed, can multiply positive impacts through the rest of the system by their ability to influence critical feedback loops.

Firstly, trust building between small-scale pig farmers and VT is a possible catalytic intervention. The concept model shows that increased trust and connection points assist prevention, reaction, and recovery from an ASF outbreak. In the R3: Trust loop, repeated farmer engagements with VTs and MAF increases farmer technical knowledge, fuelling investments in quality feed, improving biosecurity practices, and strengthening the use of appropriate antimicrobials. Along with repeated exchanges, the quality of the services provided by VT and MAF also strengthens trust. When increased knowledge and investments in good animal husbandry practices result in noticeably lower pig mortality rates, farmers strengthen their links with VTs and MAF, reinforcing knowledge gains and farm investments (R1 and R2).

As trust and connection points grow with MAF, small-scale pig farmers are more likely to report pig deaths and adhere to movement restrictions during outbreaks, working to decrease the mortality rate. The promotion of pig producer groups (PGs) as a possible intervention strategy can facilitate this process as they can foster adherence to group biosecurity rules, peer-to-peer learning, and lower monitoring costs for MAF (29). A high degree of group trust based on the social capital and social relationships of farmers has been shown as critical for PG success in Timor-Leste (30). Another critical aspect of whether farmers gain or lose trust in the system is the effectiveness of investments. In other words, do investments in biosecurity, infrastructure, and good animal husbandry practices prevent the acceleration of the R4 (providing hogs for cultural events), R5 (social capital), and R7 (poverty spiral) loops and enable pig farmers to “hang on” during a disease outbreak and later reinvest in pig farming? The ability to come through a shock like ASF with stock or capital for reinvestment has a positive impact on the whole system as it allows the number of hogs in the system to rebound quickly again and stabilizes the price of live pigs, allowing for a gradual reinvestment and restocking by farmers hardest hit by the disease. This suggests a public-private-partnership approach could be beneficial in not only creating win-win solutions to ensure continuing pig supplies but also to improve trust among the system actors, such as input suppliers, traders, and retailors.

Secondly, strengthening the capacity of MAF to provide effective services will further increase trust in the system. This entails having enough VTs to ensure pig producers can access applicable training, quality veterinary services, and timely information on disease outbreaks and preventative measures. The perceived and actual quality of services plays a critical role as pig farmers' trust and engagement depends on the perceived benefit of VT services (i.e., improved pig health, early detection of disease outbreaks, lower mortality rate) outweighing time and financial costs. Strengthening MAF services operates directly on feedback loops R2 (knowledge gains leading to improved pig health and biosecurity practices), which is countered by B1 (increasing costs) to determine if the R1: Farm investment loop operates in a virtuous manner which stimulates the R3: Trust loop. Increasing MAF capacity comes at a cost to the government of Timor-Leste as funds would need to be diverted from other government priorities. To ensure sustainability of MAF services and continuing activation of the R3 loop, institutional arrangements and fee gathering mechanisms that can lessen the financial burden on MAF should be investigated. Examples that could be considered (and later modeled) include PGs, Village Livestock Workers, and co-payments for VT services.

Lastly, following an ASF outbreak, support should be given to help pig producers restock their farms. Start-up loans or cash grants could be provided to small-scale pig farmers conditional upon application of farm biosecurity practices. In this system, the strong demand for live pigs for cultural practices may keep the price of restocking pig farms beyond the financial ability of the poorest small-scale farmers, particularly those who exhausted household savings due to the presence of the R4: Providing hogs for cultural purposes loop. Even when the B2 loop is activated, and farmers reduce their participation in cultural practices this may further exacerbate the R7: Poverty Spiral loop as the R5: Social capital loop may have caused a reduction in household savings. Providing microloans or cash grants to restock pig farms could help to stabilize live pig prices, lower the costs of cultural practices, and steady social capital stocks. Importantly, loans or grants would also ensure the B3: Restocking loop is activated, increasing the scale and diversity of small-scale farmers who re-engage in pig farming. If these loans or grants are made conditional upon investments in biosecurity practices and attendance at VT training, they would lower the susceptibility of the pig industry to future disease shocks and help activate the R3: Trust loop. Microfinance loans have been criticized for delivering modest pro-poor outcomes, potentially causing over-indebtedness, and delivering mixed performance in the SME sector (31). Moreover, the unsuitability of many MFI loan products to the agriculture sector is often highlighted, citing short loan terms that do not synchronize well with farm production cycles and regular repayment schedules that preclude borrowers from undertaking investments in lumpy assets (32). Different financial products should therefore be investigated and modeled for their impact on the system, including letters of credit, standby loans, and graduated/deferred interest loans that allow farmers to maintain positive cashflows, the latter of which are particularly critical given the high set-up and production costs and lengthy production cycles inherent to pig farming (20). The lengthy production cycle of pigs may result in continued price rises that could potentially harm farmers who did not access these credit facilities. The impact of microcredit across different farmer archetypes could be further tested by developing a quantitative SD model and comparing microcredit against other restocking options, such as importing breeding stock from neighboring regions.

### Blended SGMB Process

The blended offline and online nature of the SGMB workshops necessitated by travel restrictions was unique to this study and several lessons emerged that can be applied to similar processes in the future. The offline, tactile SGMB tools encouraged strong levels of participation from a diverse set of stakeholders and information surfaced in discussions which was new and pertinent to the MAF team. SGMB exercises follow in the rich vein of easy-to-understand participatory rural appraisal (PRA) methods (33) with the aim of drawing multi-layered contextual knowledge and facilitating robust discussions that change the mental models of participants (7). The research showed that following online training sessions that focused on theory with multiple offline opportunities to practice helped build the confidence of MAF staff to use these new tools. Early in the process the research team discussed moving participatory exercises to a full online approach; for example, using Vecta for the Layerstack exercise. This was trialed during the training of the SGMB team and slow internet speeds, intermittent loss of power and connectivity, and the unfamiliar nature of the tools combined with feedback from MAF and MSHR staff led to the development of a blended approach: offline for workshop participants but online for coaching and support of the facilitation team.

The use of two video links helped the remote process coaches guide the facilitation of the SGMB exercises. The broad video link capturing the dialogue and interactions amongst workshop members helped gauge the level of participation and acceptability of the tools (i.e., who was participating, was there active dialogue around key points, were any group members excluded?). Meanwhile, the focused video link was controlled by the translator/liaison, meaning it could be directed to an area of interest in the workshop (i.e., a conceptual model) at the discretion of the process coaches.

While there was little hindrance in remote workshop observation (beyond occasional internet black outs), it proved more challenging for remote process coaches to interject and help steer the workshop in real-time. This was partly due to the time delay in relaying messages through the translator/liaison to the workshop facilitator as well as the language barrier of communicating between Tetum and English. The translator/liaison role was essentially overloaded as the individual had to perform multiple tasks: videorecording the session, translating the workshop dialogue from Tetum to English, communicating with the two process coaches, and then digesting messages to then help coach the facilitator or ask a question to the plenary. As workshops extended into the 5-h mark, this became an exhausting process. For future virtual workshops, it would help to have one member of the facilitation team act as a pure translator who also managed the second video link and then an additional individual as the liaison between the process coaches and the facilitator.

The SGMB workshop exercises consistently went over time and the last exercise from workshops one and two had to be moved to the following session. This shortened the time available to develop concept modules (the final output of the workshops) and did not allow review and consolidation of the concept modules by the modeler between workshops two and three. Delays during the workshop could be overcome through better workshop preparation (having all resource material ready) and less repetition of exercise explanations. However, the nature of virtual process coaches and a first-time facilitation team meant delays were, to an extent, unavoidable. For example, compared to face-to-face facilitation, cues such as body language and participation levels could not be as quickly interpreted, and translations and explanations had to pass through an additional channel (the translator/liaison). Future processes should allow for the additional time required for a blended workshop approach and contain additional workshop sessions. Extra spacing between workshops would also help ensure that the large volume of workshop information collected could be translated and analyzed between workshops and further team members (beyond the lead facilitator) could have an opportunity to prepare with the process coaches. Another option would be to reduce the amount of material covered in each session, having more frequent but shorter duration workshops of 2–3 h. This latter option would have also helped prevent participant fatigue and the higher dropout rates when workshops are spread over several weeks.

The advantages of SGMB over conventional GMB highlighted in this research mirror those observed in Rich et al. (9) in Tanintharyi, Myanmar and Bihar, India. While space is an important distinguishing component and area of added value in SGMB, there are important features of the SGMB facilitation process that streamline the gathering of information and highlight patterns and associations that standard GMB would likely not. For example, the ability of stakeholders to attribute and discuss trade patterns, the evolution of disease outbreaks, and the socio-economic impacts of ASF was enhanced by SGMB. Conventional GMB exercises could eventually draw out this information, but the use of a spatially-mediated tools (like Layerstack) allows that information to be collected at the onset of the workshop so that all participants have a common understanding of the setting which is used as a shared reference in the later model building exercises. From a model building standpoint, the SGMB process, by modularizing system attributes based on space, allowed a richer and more efficient means of model conceptualization, which given the online means of facilitation saved both resources and time. The research team would have liked to probe deeper on the spatial drivers of disease, marketing, and social phenomena, over and beyond what was reported in this paper. The balancing of working with a new in-country team with no previous SD or modeling experience and the newness of all participants to conducting the training and workshop online pre-empted the full potential of the technique. Even with these limitations, the research demonstrated that spatial tools, like Layerstack, can successfully be adapted and used in a blended offline/online setting to generate the information required for fit-for-purpose models.

A number of limitations within this research should be noted as they impact the model's results and applicability to the wider pig industry in Timor-Leste. Literature suggests that participatory processes can be biased toward community members who already wield power (34), prove exclusionary to the marginalized (35), and mask invisible problems and power imbalances (36). The negative impact of power differentials between participants on GMB outcomes is also well documented (7, 37). In this research there were power imbalances amongst participants and between participants and facilitators and this could have inhibited open discussion and dialogue in SGMB sessions. Male participants outnumbered female participants and tended to dominate discussions and in some exercises active participation was limited to a smaller subset of attendees. While SGMB facilitators took steps to encourage all participants to contribute to discussions, the research could have broken into smaller group sessions (three to five participants) and increased female representation in both participants and facilitators to help mitigate gender and power imbalances like in previous GMB studies (24) and prevent “group-speak” (25). Additionally, an experienced gatekeeper embedded in the workshop could have paid attention to this and encouraged broader involvement or transmitted any concerns or questions quickly to the rest of the SGMB team (7). Lastly, participants were selected for the research through the networks of VT associated with MAF. This limited the representation of VC actors in the study and potentially swayed the prioritization of problems.

## Conclusion

This first-time application of a blended, hybrid online/offline SGMB process in Timor-Leste resulted in a rich conceptual model of the socio-economic and livelihood impacts of ASF in Timor-Leste. While there were additional challenges from the virtual nature of training, coaching, and session facilitation, the resultant model highlighted critical feedback loops which explain system behavior during an ASF outbreak that other animal health impact assessments have not explored. This led to the identification of potential leverage points for intervention by the government of Timor-Leste and development partners.

The next step in the process is to share the concept model for feedback with stakeholders in Timor-Leste, including SGMB participants and MAF staff to ensure the current structure (stocks, flows, feedback loops) accurately represents the system. The concept model was developed using Tasi Tolu as the model boundary, meaning the model and leverage points are influenced by the peri-urban context, i.e., improved access to services, and a restricted group of VC stakeholders. The verification and feedback process would require a wider group of stakeholders to agree that the model and scenarios are representative of the wider country. Once consensus is reached on the basic structure, the concept model could be expanded into a full quantitative SD model. This would require parametrization of the model variables and additional structure to support scenario-testing. As showcased in the paper, the qualitative concept model provides insights into possible leverage points in the system; however, a quantitative SD model would allow a fuller range of scenario-testing of potential interventions and trade-off analysis in terms of impacts across VC actors, time horizons, and resource constraints (i.e., financial and human capital). For example, the impact of start-up loans/cash grants on small-scale farmers could be compared against investments in training and expansion of VT or the introduction of charges for VT services. This would enable a cost-benefit analysis of standalone interventions along with intervention combinations to investigate multiplier effects. A quantitative model would also provide insight into potential negative consequences of interventions, or trade-offs that might exist across the VC nodes or impact dimensions (e.g., economic vs. equity) (10, 17, 21, 26).

The concept model presented in this paper and a future quantitative model could be readily adapted to other areas of investigation in Timor-Leste as further modules are developed and linked. For instance, the household cashflow model can be expanded so that links between investments in the pig VC and household expenditure on healthcare, education, and nutrition can be considered as part of the decision-making on intervention options. Once a robust SD model of the socio-economic and livelihood impacts of ASF in Timor-Leste is constructed and validated, it can be adapted to other contexts and requirements. The high economic and cultural value placed on pigs (38) and the recent outbreak of ASF means the model could be used to similar effect in Papua New Guinea. In countries where ASF is not yet present, such as the Solomon Islands, the model could be adapted to help understand the cost-benefit of various prevention mechanisms.

## Data Availability Statement

The raw data supporting the conclusions of this article will be made available by the authors, without undue reservation.

## Ethics Statement

The research received ethical clearance from the University of Queensland Institutional Research Ethics Committee (approval number 2020001543). Informed consent was obtained from all human subjects. The patients/participants provided their written informed consent to participate in this study. Written informed consent was obtained from the individual(s) for the publication of any potentially identifiable images or data included in this article.

## Author Contributions

TC, DS, KD, and KR conceived the study. JB, KD, and KR trained the research team and developed the methodology and analyzed the data. AP, JB, JoB, KD, KR, and OM collected the data. JB wrote the first draft of the manuscript. AP, DS, JoB, KD, KR, OM, and TC reviewed and edited the final manuscript.

## Funding

The research was undertaken within the ACIAR-LS/2019/187 Small Research Activity, Developing a Regional African Swine Fever Socioeconomic and Livelihood Impact Assessment Framework, funded by the Australian Government's Australian Centre for International Agricultural Research.

## Conflict of Interest

The authors declare that the research was conducted in the absence of any commercial or financial relationships that could be construed as a potential conflict of interest. The reviewer TSB declared a shared affiliation, though no other collaboration, with several of the authors TC and DS to the handling editor. The reviewer TSB declared a past co-authorship with one of the authors OM to the handling editor.

## Publisher's Note

All claims expressed in this article are solely those of the authors and do not necessarily represent those of their affiliated organizations, or those of the publisher, the editors and the reviewers. Any product that may be evaluated in this article, or claim that may be made by its manufacturer, is not guaranteed or endorsed by the publisher.
