# Supplementary material for: Investigating the Socio-Economic and Livelihoods Impacts of African Swine Fever in Timor-Leste: An Application of Spatial Group Model Building
Source: Front Vet Sci. 2021 Nov 18;8:687708. doi: 10.3389/fvets.2021.687708 (PMC8636599; doi:10.3389/fvets.2021.687708)
Supplement: Supplementary file 1 [file Data_Sheet_1.docx]

Supplementary Material

# Supplementary Material A: SGMB Agenda

- 1. **SGMB Workshop One Agenda**

Workshop Objectives: - Participants understand SGMB process and expected outcomes

- Use Layerstack to understand spatial dynamics of pig VC and ASF

- Identify and prioritize problems resulting from ASF outbreak

| **Length** | **Time** | **Public Agenda** | **Team Agenda** | **Preparation and Materials** |
| --- | --- | --- | --- | --- |
|  | | **PRE-WORKSHOP** | |  |
| 10 | 8.30 – 9.15 am | Arrival and registration | - Prepare room: walls free from distraction, water and snacks on desks, books and pens on desks, Layerstack ready  - Register participants and supply with name tags (colour of tag representing participant’s role in the VC) | - two types of markers, pen and a notebook for each participant  - water and snacks  - name tags, with different colours by role in the VC  - registration sheet for participants |
| 15 | 8.30 – 9.15 | Research information sharing and consent gathering | - Distribute participant information sheet  - Explain and clarify purpose of research and conditions of consent  - Distribute, witness, and gather signed consent forms  - Fill in demographic data for each participant | - Tetum translation of participant information sheet  - Tetum translation of informed consent sheet  Demographic data sheet |
|  | | **WORKSHOP** | |  |
| 5 | 9.15 | 1. Formal welcome and introductions | - General welcome and introduction of Model Building Team |  |
| 5 | 9.20 | 2. Overview of the ASF-SELIA project and purpose of SGMB workshops | - Overview of the ASF-SELIA project, covering timeline, key outcomes, and relationship to SGMB workshops  - Overview of SGMB process, including three workshops and one reflection/dissemination meeting, timing, and expected level of participation  - Reflection on goals of the process in general and consensus provided on such goals (i.e. “what’s in it for the participants?”) |  |
| 5 | 9.25 | 3. Overview of today’s workshop | - Explain sessions and how long will be spent on them  - Explain lunch details and location of bathrooms as well as Covid-safe procedures | - prepare outline of public agenda on large paper in Tetum and stick on wall so it is visible to all participants |
| 30 | 9.30 | 4. Hope and fears | - Explain that each participant needs to identify one hope and one fear they have for the workshop  - Each participant writes hopes and fears on two separate coloured cards (large clear writing) (5 mins)  - Participants individually stand in front of group, introduce themselves, and briefly explain their one hope and one fear  - Hopes and fears cards are stuck on whiteboard and collated/clustered into similar groups by assistant facilitator  - Facilitator summarises and reads out to group common themes and addresses any concerns from participants | - two types of coloured cards  - tape for sticking cards on whiteboard/wall |
| **10.00 am (15 minutes) BREAK (morning tea)** | | | | |
| 120 | 10.15 | 5. Layerstack exercise to understand pig VC and impacts of ASF | - Introduce Layerstack, showing maps, acetates, markers, and stickers  - Explain that “we are going to draw the pig VC and impacts of ASF on the various layers to help us understand the system”  - Allow 15 minutes for each layer, this includes time for facilitator to summarise key information before moving on to the next layer  - Ensure there is discussion and active participation, noting down any disagreements  Layer 1: Where are the main pig production zones in the research area? Ask about different farm sizes, mix of farming types (farrow-to-finish, wean-to-finish), breed of pigs. Any changes in the last five years? How did the ASF outbreak impact this?  Layer 2: What are the key inputs for pig production and where do they come from? Piglets, breeding sows, feed, veterinary services, credit, labour, etc.? Where and when are pig farmers/veterinarians using antibiotics on pigs? Any changes in the last five years? How did the ASF outbreak impact this?  Layer 3: Where do pigs (or piglets) from the pig production zones move to? What are pigs used for? Other villages, butchers, wholesalers, traders, retailers? Changes in demand, prices during the year? Are there seasonal patterns to movements? Any changes in the last five years? How did the ASF outbreak impact this?  Layer 4: What are the other main livelihoods in the target area? How do livelihoods contribute to household income and socio-economic status?  Layer 5: What are the impacts of ASF on these livelihoods, where are these the highest? How did ASF spread over time? Impacts on pig production, but also socio-cultural activities, and socio-economic circumstances of households? Why are some locations more affected than others? What and where are some of the wider impacts of ASF. | - A2 or A3 map of research area printed out on laminated paper  - Acetates (clear plastic layers), whiteboard markers, and stickers |
| **12.15 (10 minutes) BREAK** | | | | |
| 30 | 12.25 | 6. Problem prioritisation | - Remind participants of problems in the pig VC resulting from ASF which were identified through the Layerstack exercise  - Participants individually write down one key problem on coloured cards and then present to group, answering any questions from plenary  - Problem cards placed on whiteboard  -Compile problem cards into common themes and asks if any problems are missing  - Write additional problems on cards and places on whiteboard  - Participants are given three stickers and vote for top problems by placing stickers (maximum two per card) on problem cards  - Count voting and presents top two problems to the group, while confirming nature of problem | - coloured cards  - stickers |
| 5 | 12.55 | 7. Wrap-up | - Summarise workshop and thank participants  - Remind participants of the next workshop |  |
| **1.00 pm LUNCH** | | | | |

- 1. **SGMB Workshop Two Agenda**

Workshop Objectives: - Introduce basic System Dynamics (SD) concepts and terminology

- Complete cause and consequence mapping of problems

- Identify common issues and themes to become modules

- Draft concept modules (up to three)

| **Length** | **Time** | **Public Agenda** | **Team Agenda** | **Preparation and Materials** |
| --- | --- | --- | --- | --- |
|  | | **PRE-WORKSHOP** | |  |
| 15 | 8.00 8.15 am | Arrival and registration | - Prepare room: walls free from distraction, water and snacks on desks, books and pens on desks, Layerstack ready  - Register participants and distribute name tags (colour of tag representing participant’s role in the VC) | - two types of markers, pen and a notebook for each participant  - water and snacks  - name tags  - registration sheet for participants |
|  | | **WORKSHOP** | |  |
| 10 | 8.15 | 1. Formal welcome and overview of the day | - General welcome, including recap of previous workshop and agenda for the day | - prepare outline of public agenda on large paper in Tetum and stick on wall so it is visible to all participants |
| 40 | 8.25 | 2. Problem prioritisation | - Remind participants of problems in the pig VC resulting from ASF which were identified through the Layerstack exercise  - Participants individually write down one key problem on coloured cards and then present to group, answering any questions from plenary  - Problem cards placed on whiteboard  - Compile problem cards into common themes and asks if any problems are missing  - Write additional problems on cards and places on whiteboard  - Participants are given three stickers and vote for top problems by placing stickers (maximum two per card) on problem cards  - Count voting and presents top two problems to the group, while confirming nature of problem | - coloured cards  - stickers |
| 20 | 9.05 | 2. Introduction of SD concepts and terminology | - Introduce terminology of stocks, flows, and converters as common tools we will use to describe the pig VC and impacts of ASF  - Highlight terminology with soda-in-bottle example, show soda as stock and show water flowing in and out of bottle which changes to stock  - Add converters to change flow rate  - Draw behaviour over time graph of water in bottle  - Ask for examples of stocks, flows, and converters in the pig VC | - soda bottle  - cup  - whiteboard and markers |
| 95 | 9.25 | 3. Cause and consequence mapping of priority problems | - Review two prioritised problems  - In plenary discuss nature of problem # 1 and draw behaviour over time graphs in centre of whiteboard to describe key characteristics or changes  - Individuals write causes and consequences of problems on coloured cards  - Individual participants place ‘cause’ cards on left side of reference node. F and AF sort causes into common themes and adds further ‘causes’ and draws in relationships by asking the prompt question, “What causes this?”  - Repeat for consequences on right side of reference node, but using the key question, “Then what happens?”  - Ask participants to identify relationships between problem consequences to problem causes, documenting polarity (+ or -) of relationships and key feedback loops  - Summarise final version of cause and consequence maps and reference nodes  - Repeat for problem #2 | - whiteboard  - coloured cards for cause and effects  - markers  - tape |
| **11.00 (15 minutes) BREAK (morning tea)** | | | | |
| 20 | 11.15 | 4. Decide on modules for development | - Summarise common issues and themes that emerged across cause and consequence maps  - Group discussion on which modules should be included in the model to ensure common themes and issues are accounted for | - whiteboard |
| 90 | 11.35 | 5. Develop concept modules | - Explain that we will now develop concept modules using SD terminology of stocks, flows, and converters  - Select one module for group practise  - Identify key stocks in the system (including AMR) build structure with questions on “What causes this stock/variable to increase or decrease?”, “When this happens what is the impact?”, ensure to include polarity of relationships (i.e. + or -). Use whiteboards or large paper to sketch concept module  - Break into two small groups to develop other concept modules (30 minutes)  - Each group presents concept module to other group and updates through markers or post-it notes. | - whiteboards  - large paper  - markers  - post-it notes |
| 5 | 12.55 | 6. Wrap-up | - Summarise workshop and thank participants  - Remind participants of the next workshop |  |
| **1.00 pm LUNCH** | | | | |

- 1. **SGMB Workshop Three Agenda**

Workshop Objectives: - Review basic System Dynamics (SD) concepts and terminology

- Draft concept modules (up to four)

| **Length** | **Time** | **Public Agenda** | **Team Agenda** | **Preparation and Materials** |
| --- | --- | --- | --- | --- |
|  | | **PRE-WORKSHOP** | |  |
| 30 | 8.30 – 9.00 am | Arrival and registration | - Prepare room: walls free from distraction, water and snacks on desks, books and pens on desks  - Register participants and distribute name tags (colour of tag representing participant’s role in the VC) | - two types of markers, pen and a notebook for each participant  - water and snacks  - name tags  - registration sheet for participants |
|  | | **WORKSHOP** | |  |
| 15 | 9.00 | 1. Formal welcome and overview of the day | - General welcome  - Recap of previous workshop and key learning  - Share agenda for the day | - prepare outline of public agenda on large paper in Tetum and stick on wall so it is visible to all participants |
| 20 | 9.15 | 2. Review terminology of SD concepts and terminology | - Introduce terminology of stocks, flows, and converters as common tools we will use to describe the pig VC and impacts of ASF  - Ask for examples of stocks, flows, and converters in the pig VC and draw simple stock and flow diagrams of examples | - soda bottle  - cup  - whiteboard and markers |
| 10 | 9.35 | 3. Introduce concept modules for development | - Summarise common issues and themes that emerged across cause and consequence maps  - Remind participants that four concept modules were selected for development: i) Pig production; ii) Pig disease; iii) Pig farm finances; and iv) Socio-cultural connections |  |
| 90 | 9.45 | 4. Develop concept modules | - Share with participants simple concept modules developed prior to the workshop by Jared and Karl  - Explain that we will now expand these concept modules using SD terminology of stocks, flows, and converters  - Select pig production module for group practise  - Present key stocks in the system (including AMR) build structure with questions on “What causes the flow into / out of this stock to increase or decrease?”, “When this happens what is the impact?”, ensure to include polarity of relationships (i.e. + or -). Use whiteboards or large paper to sketch concept module  - Break into two small groups to develop other concept modules (30 minutes), one group continue pig production and pig disease, the other group develops pig farm finances and socio-cultural connections. | - whiteboards  - large paper  - markers  - post-it notes |
| 11.15 BREAK 20 minutes  During break lead facilitator and assistant facilitator talk through the concept modules with process coaches for advice on follow-up questions, areas to explore | | | | |
| 75 | 11.30 | 5. Review concept modules | - Each group presents concept module to larger group for feedback and updating  - Four concept modules are hung on walls of workshop and connections between modules identified and drawn in.  - Identify any feedback loops between the concept modules and determine polarity of connections. | - large paper  - markers  - post-it notes |
| 15 | 12.45 |  | - Summarise workshop and key points and thank participants  - Ask participants for any feedback on the workshop process  - Update participants on the final workshop/sharing session  - Take group picture |  |
| **1.00 pm LUNCH** | | | | |

# Supplementary Material B: Concept model individual feedback loops


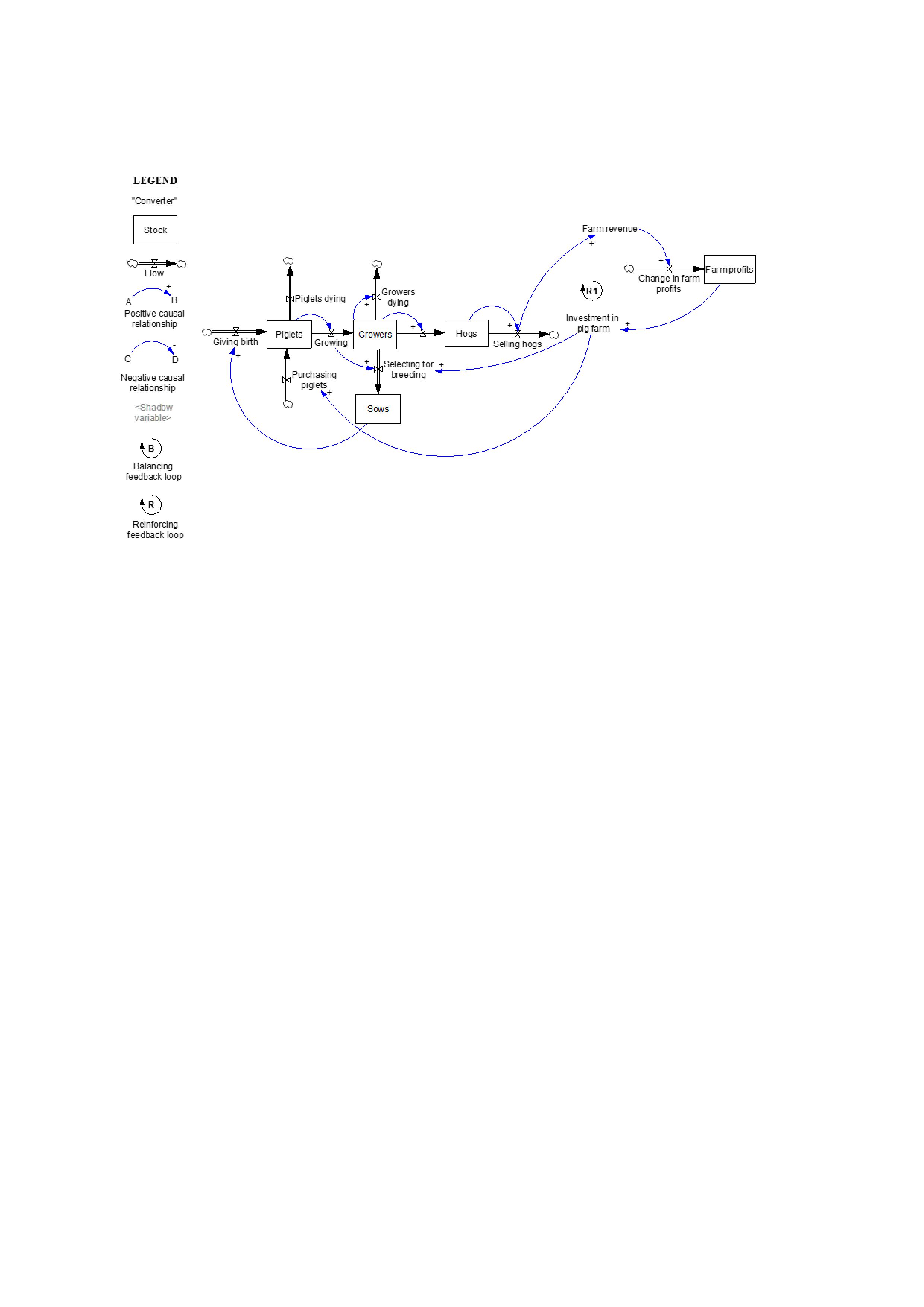


Supplementary Figure 1. Feedback loop R1: Farm Production Investments. Grey parameters are repeated “shadow” variables from the concept model. “R” indicates reinforcing feedback loops and “B” indicates balancing feedback loops in the system. Unboxed text represents key converters (also known as parameters) in the model while boxed text represents key stocks in the system and black arrows show flows into and out of these stocks. Blue arrows show critical casual relationships between stocks, flows, and converters with the + sign indicates movement in the same direction as the origin of the change and the - sign indicates movement in the opposite direction to the origin of change.


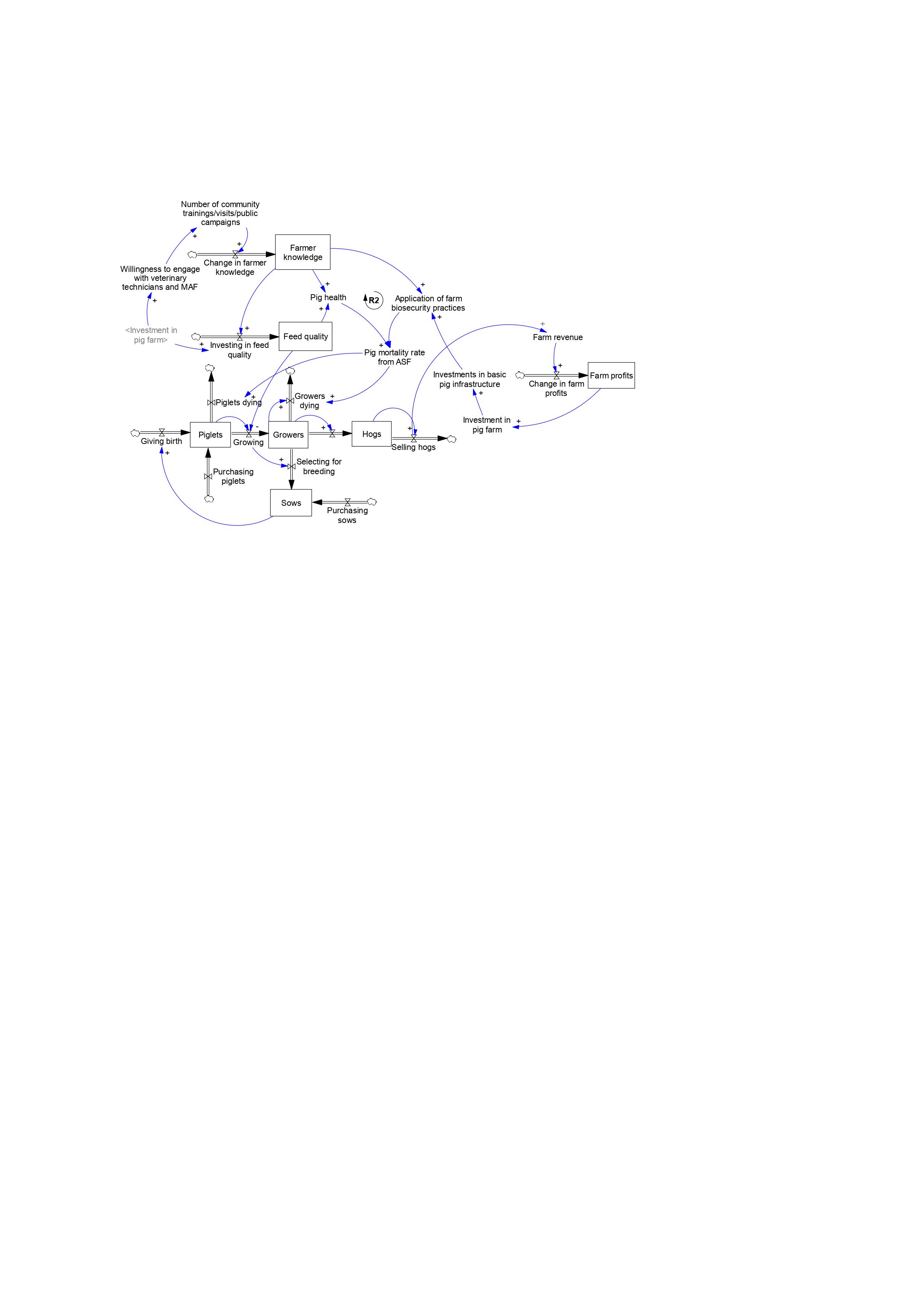


Supplementary Figure 2. Feedback loop R2: Farm biosecurity/health investments. Grey parameters are repeated “shadow” variables from the concept model. “R” indicates reinforcing feedback loops and “B” indicates balancing feedback loops in the system. Unboxed text represents key converters (also known as parameters) in the model while boxed text represents key stocks in the system and black arrows show flows into and out of these stocks. Blue arrows show critical casual relationships between stocks, flows, and converters with the + sign indicates movement in the same direction as the origin of the change and the - sign indicates movement in the opposite direction to the origin of change.


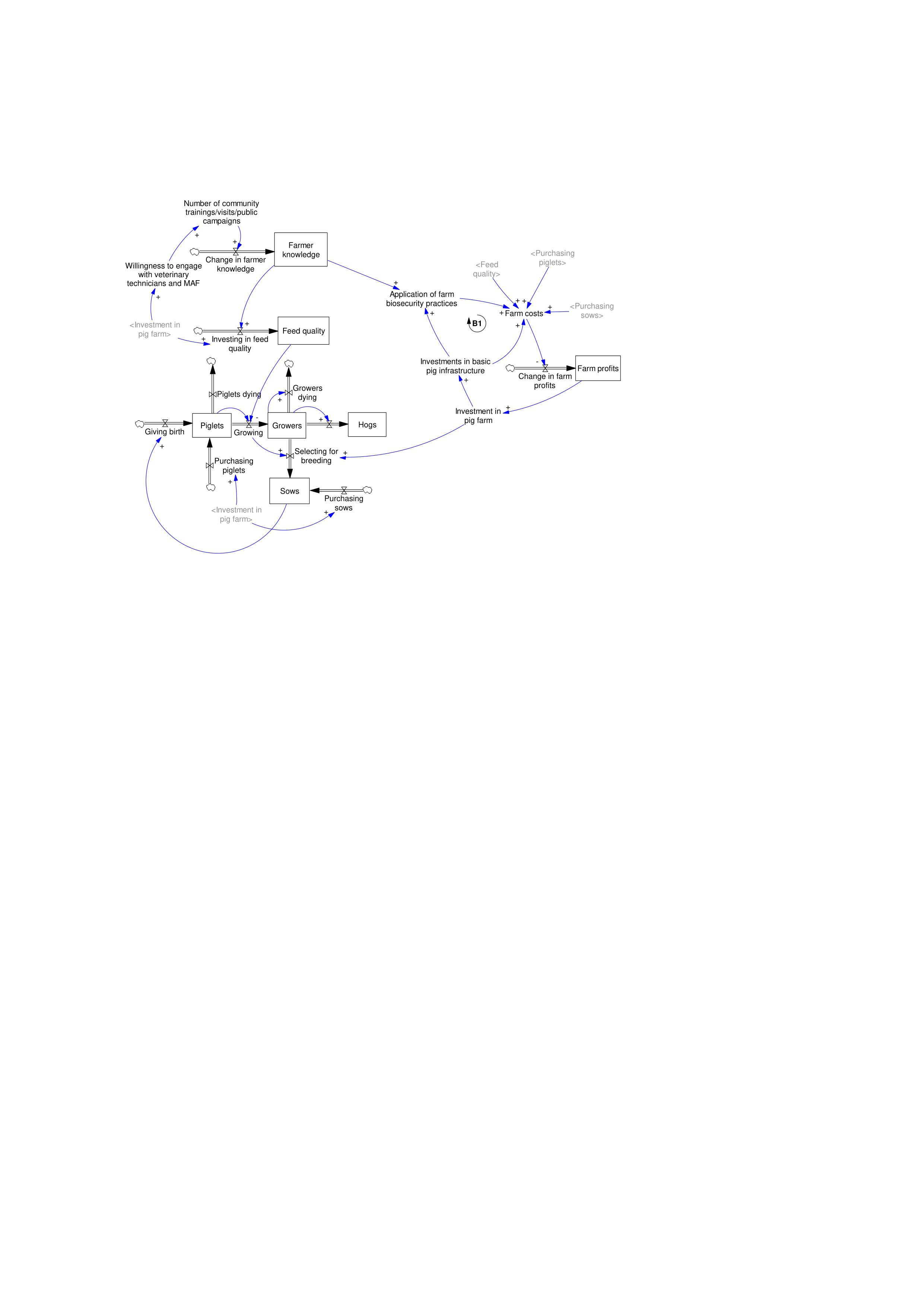


Supplementary Figure 3. Feedback loop B1: Farm costs. Grey parameters are repeated “shadow” variables from the concept model. “R” indicates reinforcing feedback loops and “B” indicates balancing feedback loops in the system. Unboxed text represents key converters (also known as parameters) in the model while boxed text represents key stocks in the system and black arrows show flows into and out of these stocks. Blue arrows show critical casual relationships between stocks, flows, and converters with the + sign indicates movement in the same direction as the origin of the change and the - sign indicates movement in the opposite direction to the origin of change.


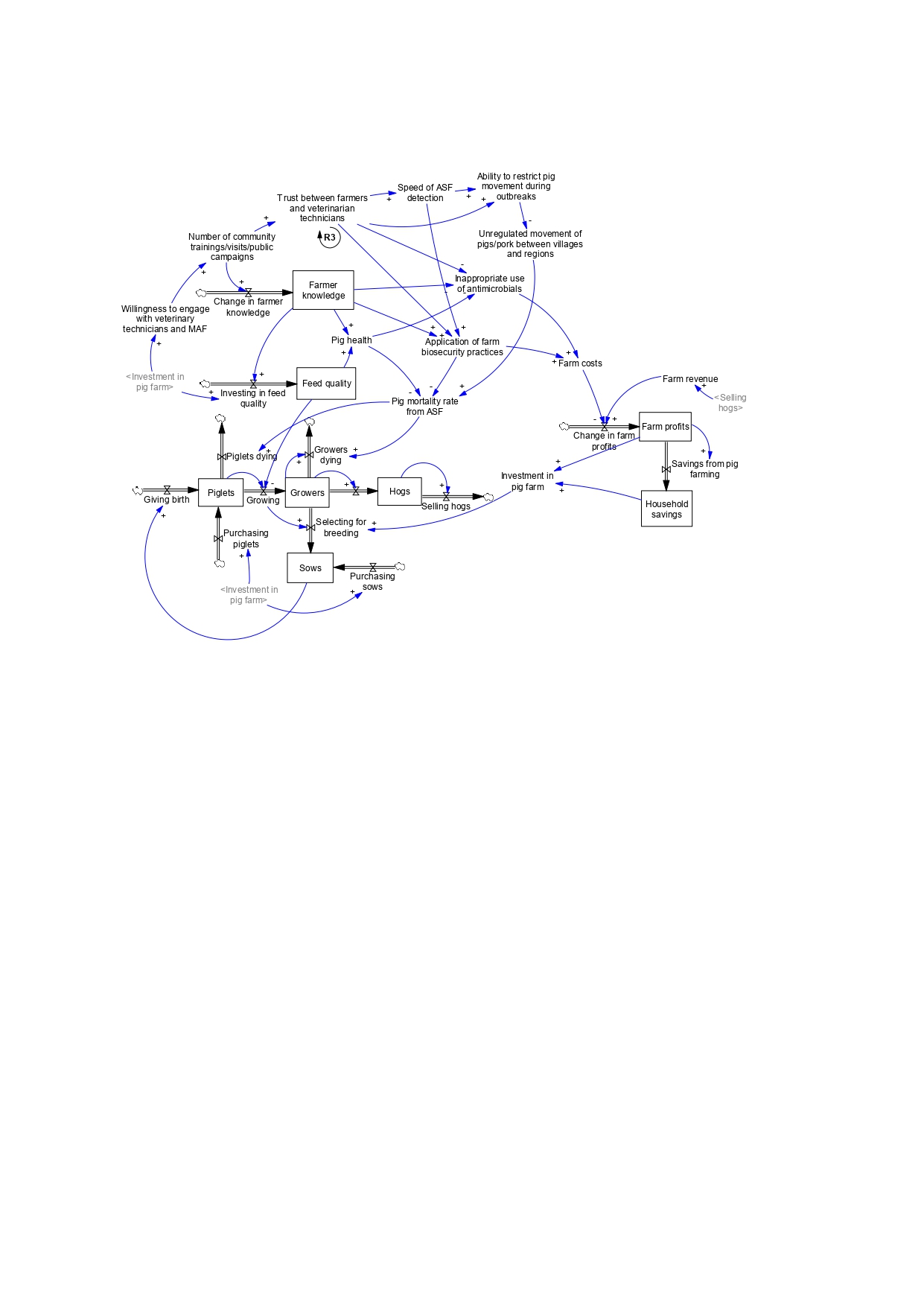


Supplementary Figure 4. Feedback loop R3: Trust. Grey parameters are repeated “shadow” variables from the concept model. “R” indicates reinforcing feedback loops and “B” indicates balancing feedback loops in the system. Unboxed text represents key converters (also known as parameters) in the model while boxed text represents key stocks in the system and black arrows show flows into and out of these stocks. Blue arrows show critical casual relationships between stocks, flows, and converters with the + sign indicates movement in the same direction as the origin of the change and the - sign indicates movement in the opposite direction to the origin of change.


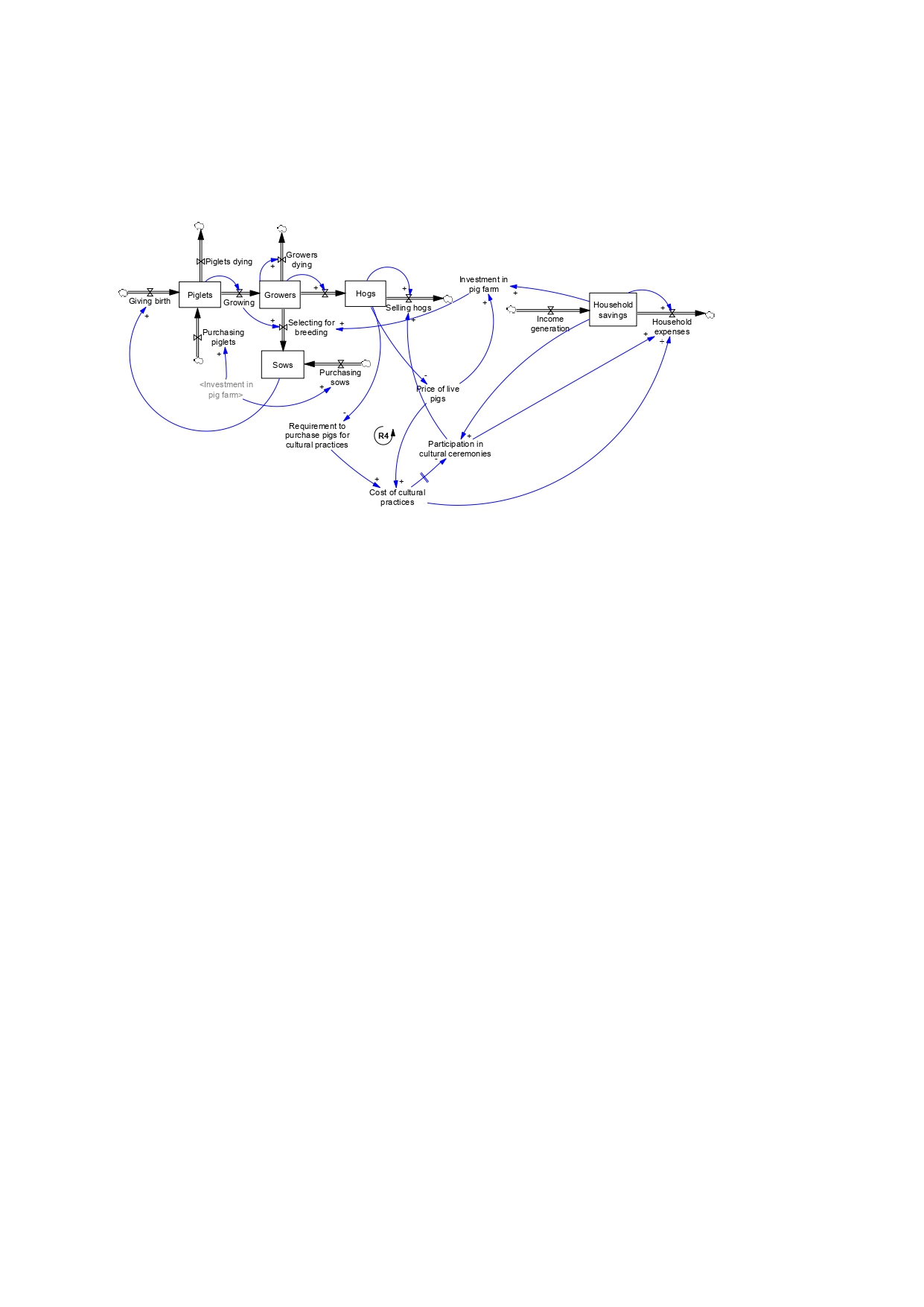


Supplementary Figure 5. Feedback loop R4: Providing hogs for cultural practices. Grey parameters are repeated “shadow” variables from the concept model. “R” indicates reinforcing feedback loops and “B” indicates balancing feedback loops in the system. Unboxed text represents key converters (also known as parameters) in the model while boxed text represents key stocks in the system and black arrows show flows into and out of these stocks. Blue arrows show critical casual relationships between stocks, flows, and converters with the + sign indicates movement in the same direction as the origin of the change and the - sign indicates movement in the opposite direction to the origin of change.


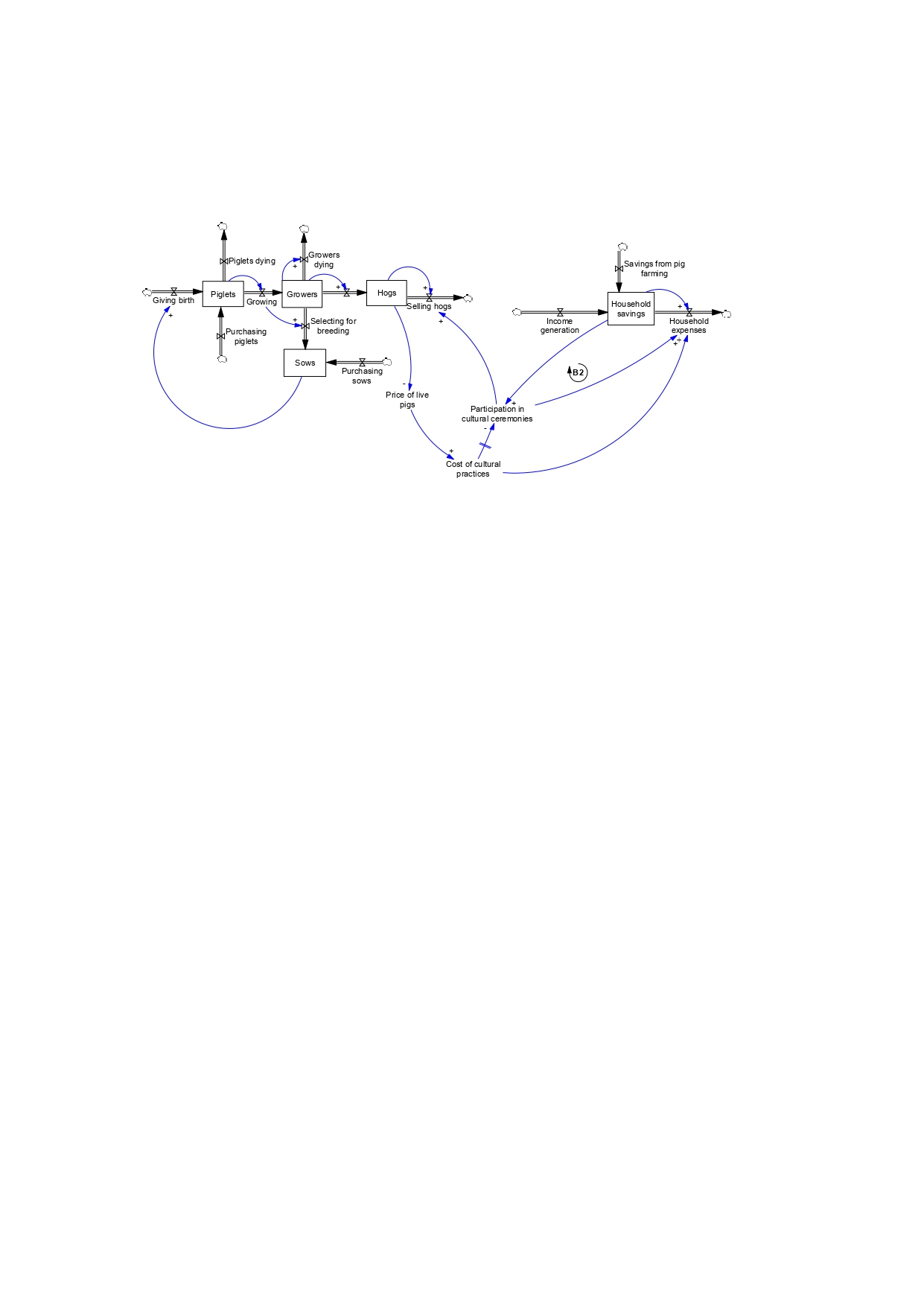


Supplementary Figure 6. Feedback loop B2: Reducing participation in cultural practices. Grey parameters are repeated “shadow” variables from the concept model. “R” indicates reinforcing feedback loops and “B” indicates balancing feedback loops in the system. Unboxed text represents key converters (also known as parameters) in the model while boxed text represents key stocks in the system and black arrows show flows into and out of these stocks. Blue arrows show critical casual relationships between stocks, flows, and converters with the + sign indicates movement in the same direction as the origin of the change and the - sign indicates movement in the opposite direction to the origin of change.


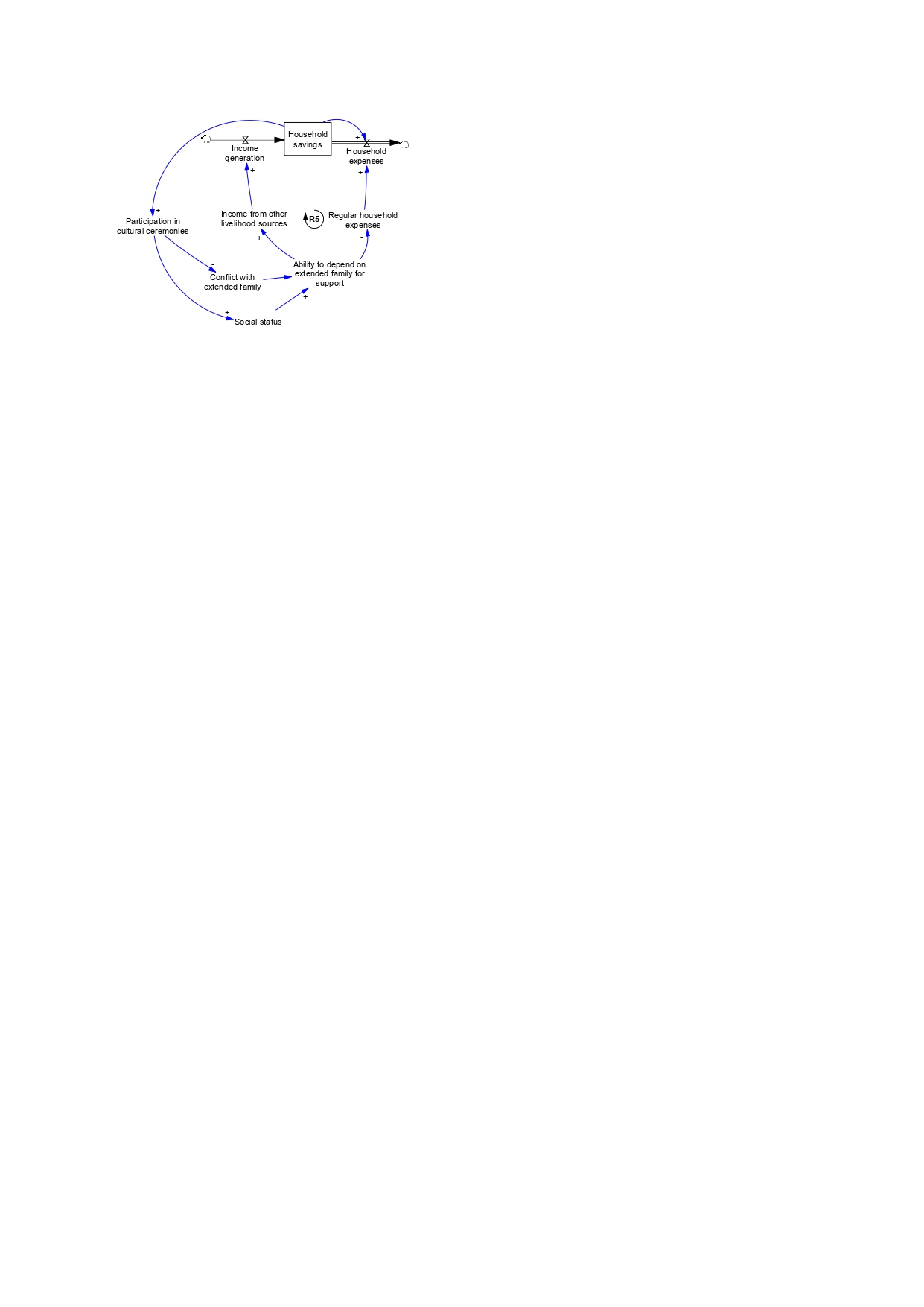


Supplementary Figure 7. Feedback loop R5: Social capital. Grey parameters are repeated “shadow” variables from the concept model. “R” indicates reinforcing feedback loops and “B” indicates balancing feedback loops in the system. Unboxed text represents key converters (also known as parameters) in the model while boxed text represents key stocks in the system and black arrows show flows into and out of these stocks. Blue arrows show critical casual relationships between stocks, flows, and converters with the + sign indicates movement in the same direction as the origin of the change and the - sign indicates movement in the opposite direction to the origin of change.


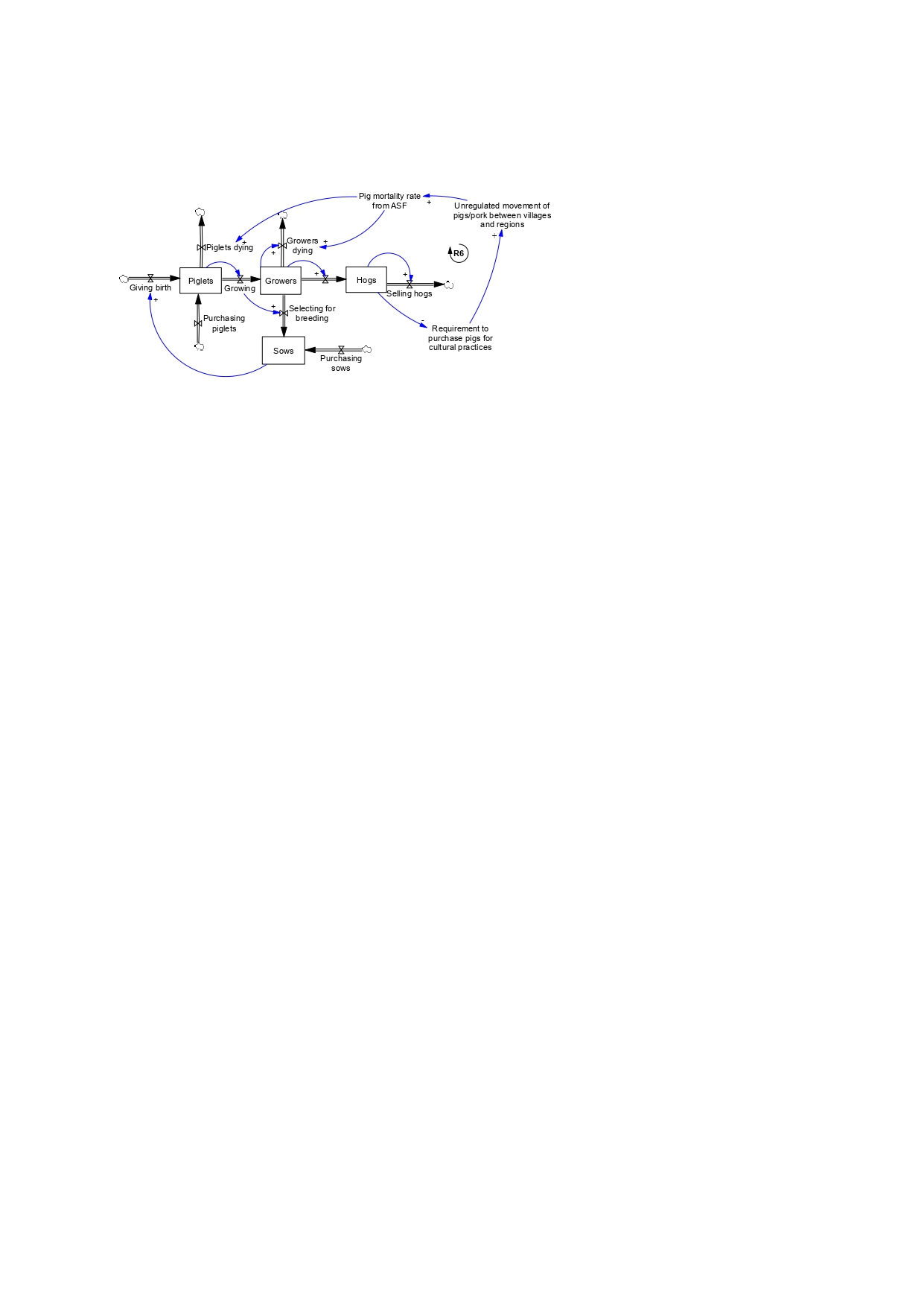


Supplementary Figure 8. Feedback loop R6: Movement of pigs. Grey parameters are repeated “shadow” variables from the concept model. “R” indicates reinforcing feedback loops and “B” indicates balancing feedback loops in the system. Unboxed text represents key converters (also known as parameters) in the model while boxed text represents key stocks in the system and black arrows show flows into and out of these stocks. Blue arrows show critical casual relationships between stocks, flows, and converters with the + sign indicates movement in the same direction as the origin of the change and the - sign indicates movement in the opposite direction to the origin of change.


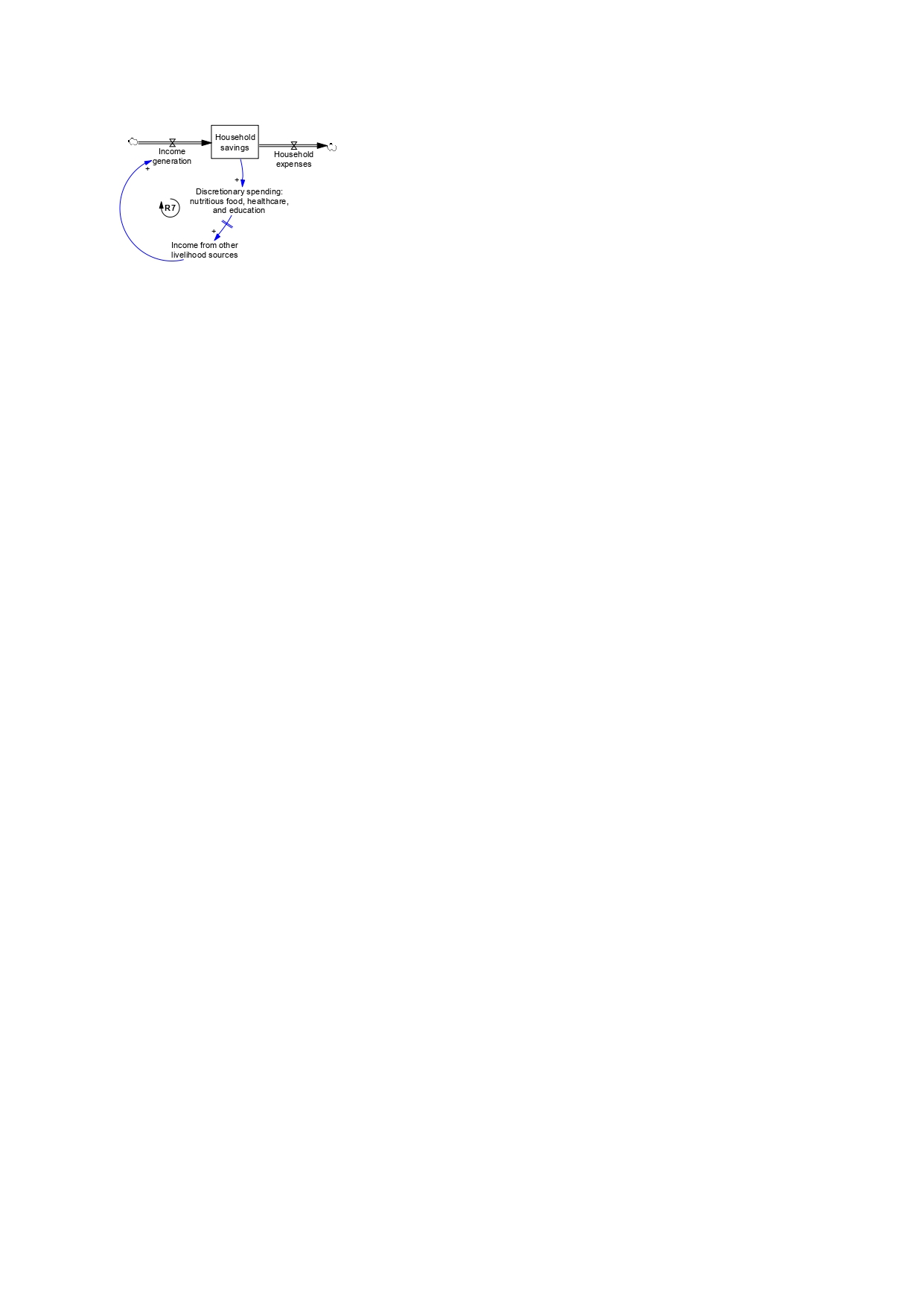


Supplementary Figure 9. Feedback loop R7: Poverty spiral. Grey parameters are repeated “shadow” variables from the concept model. “R” indicates reinforcing feedback loops and “B” indicates balancing feedback loops in the system. Unboxed text represents key converters (also known as parameters) in the model while boxed text represents key stocks in the system and black arrows show flows into and out of these stocks. Blue arrows show critical casual relationships between stocks, flows, and converters with the + sign indicates movement in the same direction as the origin of the change and the - sign indicates movement in the opposite direction to the origin of change.


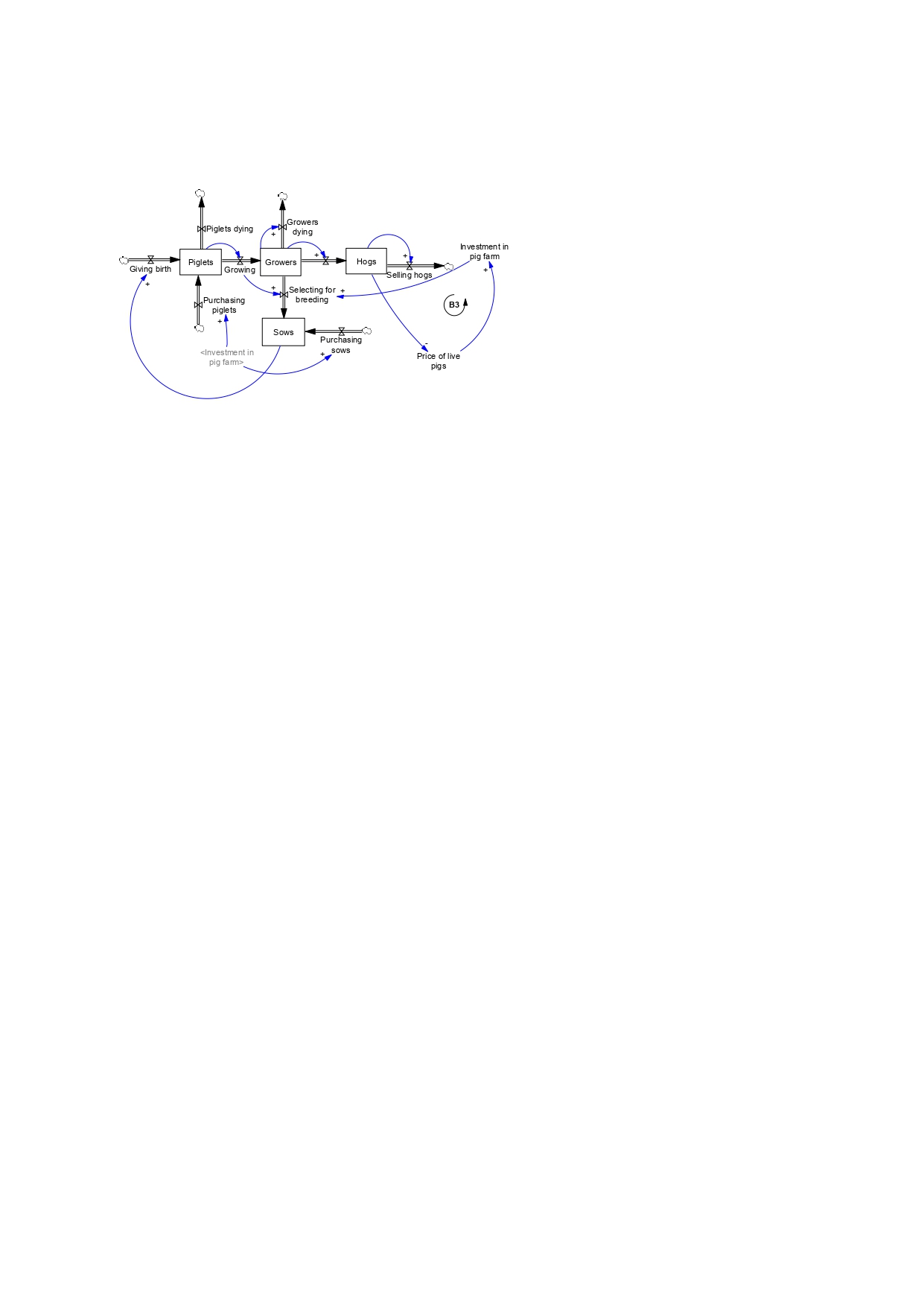


Supplementary Figure 10. B3: Restocking. Grey parameters are repeated “shadow” variables from the concept model. “R” indicates reinforcing feedback loops and “B” indicates balancing feedback loops in the system. Unboxed text represents key converters (also known as parameters) in the model while boxed text represents key stocks in the system and black arrows show flows into and out of these stocks. Blue arrows show critical casual relationships between stocks, flows, and converters with the + sign indicates movement in the same direction as the origin of the change and the - sign indicates movement in the opposite direction to the origin of change.
